# The Hardness Evolution of Cast and the High-Cycle Fatigue Life Change of Wrought Ni-Base Superalloys after Additional Heat Treatment

**DOI:** 10.3390/ma14237427

**Published:** 2021-12-03

**Authors:** Juraj Belan, Lenka Kuchariková, Eva Tillová, Miloš Matvija, Milan Uhríčik

**Affiliations:** 1 Department of Materials Engineering, Faculty of Mechanical Engineering, University of Žilina, Univerzitná 8215/1, 010 26 Žilina, Slovakia; lenka.kucharikova@fstroj.uniza.sk (L.K.); eva.tillova@fstroj.uniza.sk (E.T.); milan.uhricik@fstroj.uniza.sk (M.U.); 2 Institute of Materials and Quality Engineering, Faculty of Materials, Metallurgy and Recycling, Technical University of Košice, Letná 9, 042 00 Košice, Slovakia; Milos.Matvija@tuke.sk

**Keywords:** manufacturing engineering of material, advanced engineering material, Ni-base superalloys, heat treatment, SEM analysis, TEM analysis, phases in superalloys, Vickers hardness, fatigue test

## Abstract

Concerning the use of modern technologies and manufacturing systems in the production of high-stress components from Ni-base superalloys and the optimization of the production process, knowledge of the microstructure–mechanical properties relationship is very important. The microstructure of Ni-base superalloys is very closely related to the chemical composition. With the high number of alloying elements, various phases are presented in the structure of Ni-base superalloys, which have a predominantly positive effect on the mechanical properties, but also phases that reduce, in particular, the heat resistance of these materials. The aim of the presented paper is the quantification of structural parameters of two types of cast alloys, ZhS6K and IN738, where the effect of dwell at 10 and 15 h at 800 °C on the change in morphology and volume fraction of the γ′-phase precipitate was studied. The detected changes were verified by the Vickers hardness test. The IN718 superalloy was chosen as a representative of the wrought superalloy. This alloy was also annealed for 72 h at a temperature of 800 °C, and the quantification of structural parameters was performed by EDS mapping and TEM analysis. Another partial goal was to assess the effect of changes in the volume fraction of the γ′-phase and δ-phase on the change in the high-cycle fatigue life of superalloy IN 718. This superalloy was tested by dynamic cyclic loading with cycle asymmetry parameter R = −1 at an ambient temperature of 22 ± 5 °C and at a temperature of 700 ± 5 °C and with cycle asymmetry parameter R < 1 (three-point bending load) after annealing at 700 °C/72 h. The results of the quantitative analyses and fatigue tests will be further used in optimizing the design of Ni-base superalloy components by modern technologies such as additive technologies for the production of turbine blades and implemented within the philosophy of Industry 4.0.

## 1. Introduction

Superalloys are the materials most commonly used in environments characterized by a combination of high operating temperatures and mechanical stress. In general, they can be divided according to the basic element that forms a solid solution into three basic groups: superalloys based on nickel, cobalt, and iron. All types of superalloys are characterized by a basic matrix crystallizing in a cubic, face-centered FCC lattice with different types of precipitated phases that provide secondary hardening of the basic solid solution. However, of these superalloy-forming elements, only nickel retains the FCC lattice throughout the temperature range—it is not a polymorphic metal. Other superalloy-forming elements are polymorphic, which means that they have allotropic modifications. Iron crystallizes in the FCC lattice only between 911 °C and 1392 °C and is referred to as Feγ. Cobalt has an allotropic transformation temperature of 417 °C. Up to this temperature, it crystallizes in a hexagonal, close-packed HCP (Coα) lattice; above this temperature and up to the melting point, it has a cubic, face-centered FCC (Coβ) lattice. Since the FCC lattice has the highest stability at high temperatures compared to other lattices, ferrous superalloys are alloyed with Ni, Co, C, and Mn to stabilize the γ region. Cobalt superalloys are alloyed with Mn, Ni, and C to stabilize the β region [1].

The most widespread group of superalloys consists of superalloys based on Ni–Fe. These superalloys are characterized by high toughness and ductility. They are most often produced as molded semi-finished products, forged, and used for applications up to about 650 °C to 700 °C. The very process of forging, forming the size of the basic γ grain, can be successfully controlled to ensure the required combination of properties [2]. Concerning the method of achieving the required properties, these superalloys are divided into so-called precipitation hardened superalloys having γ′-Ni_3_(Al, Ti) or γ″-Ni_3_Nb precipitates dispersed in the basic γ matrix; superalloys with a low coefficient of thermal expansion (CTE) and specially processed and alloyed stainless steels, the required properties of which are achieved by strengthening the basic solid solution γ with finely dispersed carbide particles.

The category γ-phase precipitation hardened superalloys include, for example, A-286, V-57, Nimonic 901, and Inconel 718. However, γ′-phase precipitation in these Ni–Fe alloys is due to the low aluminum content. It follows that to increase the volume of the strengthening γ′-phase, the addition of Ti is required, which then forms a γ′-Ni_3_Ti precipitate. This precipitate is stable up to 650 °C. At longer exposures at this temperature, γ′-Ni_3_Ti transforms into a coarse, plate-deposited η-Ni_3_Ti phase, which crystallizes in a hexagonal, close-packed lattice (HCP Ni_3_Ti). Several works deal with the transformation of the γ′-Ni_3_Ti phase to η-phase [3,4,5]. The η phase occurs in Ni, Ni–Fe, and Co alloys, where the ratio of Ti:Al alloying elements is >1, most often about 3:1. It is formed at higher temperatures or longer heat exposure above about 750 °C when the metastable Ni_3_Ti γ′-phase coarsens and dissolves—thus, there is a gradual loss of coherence with the γ matrix. For this reason, the DO_24_ η-phase was considered to replace the γ′-phase as a reinforcing phase at higher temperatures. The η phase is most often excreted at grain boundaries, either in the form of blocks or needles at aging temperatures in the range of 600–850 °C or with a Widmanstätten morphology at aging temperatures above 850 °C [5]. Some studies suggest that the η-phase may also arise as a consequence of the degradation of primary MC carbides, as their degradation releases Ti [6,7]. Further studies have described that the η-phase can also occur at the expense of the γ′-phase because the η-phase is more stable at higher temperatures compared to the γ′-phase [5,8]. Ou et al. [9] pointed out that at higher aging temperatures (750–800 °C) and longer times, new volumes of needle η-phase are formed, nucleating preferentially at grain boundaries and around MC carbides, subsequently growing into the grain. The formation of new volumes of the η-phase is also related to the depletion of its surroundings by the γ′-phase, which is a logical consequence of the depletion of the γ′-phase by Ti to reduce the value of the stacking fault energy (SFE). The effect of Ti content and other elements on the SFE value will be discussed further. The needle morphology of the η-phase at temperatures above 750 °C acts as a stress concentrator, where dislocations accumulate, resulting in the formation of microcracks and a reduction in creep rupture life. Thus, the effect of the η-phase on the mechanical properties of Ni superalloys is related to its morphology, position, and amount. A large amount of η-phase in the form of Widmanstätten needles inside the grain usually reduces the mechanical properties by depleting the grain by the volume of the γ′-phase. However, a small amount of η-phase present in the form of blocks or cellular form may prevent the propagation of cracks and grain boundaries slipping during deformation at higher temperatures [10], and also results in a significant reduction in strength [11]. The low content of aluminum in these alloys has another negative effect: the heat resistance of the alloy is reduced due to insufficient formation of aluminides in the surface layers. In Nb-containing alloys, another form of precipitate is formed, the body-centered tetragonal γ″-phase of Ni_3_Nb. A classic example is the weldable superalloy Inconel 718, the production of which represents approximately 35% of the total production of wrought Ni–Fe superalloys [4]. This superalloy is most commonly used to make jet engine components such as compressors, turbine discs, casings, compressor blades, and fasteners. Under certain circumstances, superalloys may also contain a combination of all three alloying elements (Al, Ti, and Nb). In this case, the so-called double hardening effect due to the exclusion of both γ′/γ″-phases (for example, for Inconel 706, 709, and 718 alloys) occurs. These three alloys can also be considered as Ni-base superalloys, but they have a sufficient Fe content (as a substitute for deficient Ni) to be considered as Ni–Fe superalloys.

As stated by various authors [2,11,12,13,14,15,16,17], the microstructure of polycrystalline Ni-base superalloys consists of two fundamental components. These are ordered intermetallic phases of the type γ′ (L1_2_) or γ″ (D0_22_), which are characterized by a high degree of coherence with the basic solid solution γ (FCC). It is thanks to these intermetallic precipitates and their high coherence with the matrix γ that unique high-temperature mechanical properties are obtained. To ensure the optimal amount of γ′/γ″ precipitates, polycrystalline Ni-base superalloys are alloyed with a medium content of elements such as Al, Ti, Ta, or Nb. These elements have an atomic radius greater than Ni and therefore stabilize the Ni_3_X γ′-phase. Shi et al. [18] discussed the growth mechanism of γ′/γ″ phases. A critical temperature at which significant morphological changes occur in both the γ′-phase and the γ″-phase according to their work is 649 °C. These changes are related to the coarsening and elongation of the γ″-phase plates and the coarsening of the γ′-phase along the <001>γ′, <011>γ′, and <111>γ′, planes. All elements excluded in the γ′-phase increase the value of its SFE, as stated by Zacherl et al. [19]. They also stated that these elements (Al, Ti, and Nb) reduced the value of the diffusion activation energy and thus increase the creep rate. Other alloying elements such as Co, Fe, Cr, Ru, Mo, Re, and W, which have an atomic radius similar to Ni, are situated mainly in solid solution γ. The main alloying elements Co, Mo, W, Cr, and Fe significantly reduce the value of the γ matrix SFE, and at the same time, increase the diffusion activation energy, reducing the creep rate, and thus increasing the heat resistance of the basic solid solution; they also positively stabilize the precipitates [19,20,21].

The relationship between applied heat treatment–mechanical properties on different types of Ni-base superalloys has been discussed by several authors. The influence of different heat treatment conditions on the microstructure of the cast IN738LC polycrystalline alloy was discussed by Bagoury et al. [22]. The results of their work showed that the solution treatment consisting of a combination of 1180 °C/1.5 h + 1220 °C/2 h reduces segregation and improves the distribution and homogeneity of alloying elements in the IN 738LC alloy. Mitchell et al. [23] studied the effect of cooling rate from the temperatures above γ′-phase solvus on its morphology, lattice mismatch, and hardness in polycrystalline Ni superalloys of the RR 1000 category. They found that the high cooling rate from above γ′-phase solvus causes unimodal spherical or cubic γ′-phase precipitation. Decreasing the cooling rate, in turn, leads to the formation of a bimodal γ′-phase with a larger linear dimension. The applied cooling rates increase the strength and reduce the residual stresses also may cause the γ-γ′ lattice mismatch parameter to decrease due to γ-phase saturation with larger atomic radius elements, which preferably form the γ′-phase and which did not have sufficient diffusion energy when γ′-phase coarsening. The hardness of the polycrystalline alloys increased with increasing cooling rate and was not affected by the value of the γ-γ′ lattice mismatch. Ceena et al. [24] investigated the effect of heat treatment consisting of two-stage precipitation at 1010 °C/2 h + 788 °C/8h on the microstructure and tensile properties at room temperature of the Haynes 282 superalloy. Used heat treatment results in the discrete precipitation of carbides across the grain boundaries and the formation of a fine intergranular γ′-phase with an average size of 43 nm, which gives excellent tensile strength and ductility at room temperature. At the same time, however, they pointed out that increasing the solution annealing temperature to 1120 °C led to grain growth, and at the same time, γ′-phase coarsening (with the average size 100 nm). The coarsening of the γ′-phase and the formation of a carbide net at the grain boundaries significantly reduced the tensile strength and reduced the toughness by almost 50%. In their work, Wang et al. [25] described the effect of cooling rate (0.15 °C/s, 0.60 °C/s, 72 °C/s, and 138 °C/s) on the microstructure and high-temperature mechanical properties of the experimental monocrystalline superalloys. They found that as the cooling rate increased, the γ′-phase nucleation rate increased, resulting in an increased volume of this phase. At the same time, they described the change in γ′-phase morphology from originally cubic to spherical. These changes also had a positive effect on the mechanical properties (yield strength 0.2% YS, ultimate tensile strength UTS, and Young’s modulus E) at 980 °C. The increase in these characteristics was due to an increase in lattice mismatch, resulting in a coherent strain between the γ′-phase and the γ-phase, which caused a higher elastic stress field around the γ′-phase and hindered dislocation movement. The change in microstructure, tensile strength, and creep properties in the polycrystalline superalloy K417G is discussed in Du et al. [26] and the effect of solution annealing on the creep properties of Ni monocrystalline superalloy in Du et al. [27]. The process of the fatigue of Ni-base superalloys has been intensively studied by several researchers over the last decade. Abikchi et al. [28] discussed the effect of different grain sizes of wrought alloy IN718 on fatigue crack initiation and fatigue life at a load frequency of 1 Hz and a test temperature of 450 ± 2 °C. They concluded that, in the case of an alloy with an average grain size of 10 µm, a fatigue crack is initiated at the boundaries of these grains, which are joined in the free surface. An alloy with a grain size of about 7 µm showed a different mechanism of fatigue crack initiation, initiated on TiN particles just below the surface and forming a so-called “fish-eye”—a characteristic initiation more common in rotation bending and low loading frequencies. There is a relationship between γ″-phase size and fatigue crack initiation. The γ″-phase present in the IN718 alloy affects the homogeneity of the slip bands and thus reduces the probability of fatigue crack initiation by PSBs. In the case of a fine-grained structure, the γ″-phase size increases and the slip bands are more homogeneously distributed in the grains. In the work of Ogawahara et al. [29], the relationship between the size of the internal defects and the fatigue life of IN718 was discussed. Based on their research, it is negligible and minimally affects the fatigue life of the alloy. The effect of texture on the low cycle fatigue (LCF) life of selective laser melted (SLM) IN 718 is discussed in Zhou et al. [30]. Their research showed that an alloy with a columnar structure showed a significant anisotropy of LCF properties, while samples with a parallel structure did not. High-cycle and high-frequency (HCHF) fatigue of IN 718 alloy are discussed in [31,32]. Zhong et al. [31] describe the high-cycle fatigue (HCF) properties of the alloy IN718 in the following states: (i) solid solution state and (ii) after precipitation hardening at ambient temperature with a cycle asymmetry parameter R = −1 and a load frequency of about 130 Hz. Their findings showed that there was a minimal difference in HCF life between the state after solution annealing and after precipitation hardening (only 6.3%). Song et al. [32] described the very high cycle fatigue properties of SLM alloy IN718 at the test temperature of 650 °C, which is the critical temperature of use for the alloy IN718 due to the transformation of metastable γ″-phase to stable δ-phase, and compared the results with classical wrought alloy. They stated that the test temperature of 650 °C had an almost negligible effect on the fatigue life of the SLM alloy at the fracture number of cycles up to 10^8^, but with an increasing number of cycles, above 10^8^, the fatigue life decreased significantly. In the range of cycles up to 10^7^ ÷ 10^8^, the fatigue life of both alloys was almost the same and the fatigue crack initiation was predominantly on the surface of the alloys. The wrought alloy showed higher fatigue properties at the number of cycles to fracture above 10^8^ compared to the SLM alloy, and the initiation of the fatigue crack moved just below the surface, where it took place on internal defects or microstructural discontinuities.

In recent decades, the influence of the γ″-phase on the mechanical properties of Fe–Ni superalloys has been the subject of extensive research, both at ambient temperature and at elevated temperatures. However, research into the effect of another important phase in Fe–Ni superalloys with a higher content of Nb, δ-phase, has long remained secluded. Only more detailed studies of metallurgical and physical changes in the crystallization process of Fe–Ni superalloys have shown the importance of knowing its effect on mechanical properties in the range of application temperatures above 600 °C. Current studies regarding the δ-phase on static, and especially dynamic properties, at temperatures above 650 °C provide conflicting information about its positive, resp. negative impact. In general, current studies have not shown a significant effect of the δ-phase on increasing the hardening effect of the IN718 alloy. In contrast, as stated in [33,34], its presence reduced the yield strength and UTS strength, but at the same time, improved the toughness of the alloy. Similar conclusions were reached by Kuo et al. [35] in high-temperature tests at constant load. The study of the effect of a small amount of δ-phase (up to 1.5%) on the basic mechanical properties (YS, UTS, ductility, and hardness) was discussed in the work of Valle et al. [36]. Their research showed that with low δ-phase content, these characteristics did not change significantly, but rather affected the size of the original austenitic grain. The results of the work of Sundararaman et al. [37] declared that the formation of a larger volume fraction of δ-phase reduced the strength of superalloys strengthened by γ″-phase precipitates due to depletion of the original γ″-phase by elements involved in its formation, and at higher temperatures (above 650 °C) enter the δ-phase.

This paper aims to assess the effect of applied heat treatment on the reduction in chemical heterogeneity of cast superalloys, which is represented by the secondary dendrite arm spacing (SDAS factor) in this study and the change in hardness due to increased volume and morphology change of the γ′-phase. Since only a few studies have been carried out for the higher volume of δ-phase and fatigue properties at ambient temperature and higher temperatures, this study also aimed to determine how the δ-phase higher volume affected the fatigue properties when the IN718 alloy was subjected to push–pull symmetrical fatigue loading at very high-frequency (above 20,000 kHz) and room temperature (very high cycles fatigue loading, VHCFL), low-frequency (approximately 64 Hz) and temperature 700 °C ± 5 °C (low cycle fatigue loading, LCFL), and medium frequency (approximately 150 Hz) fatigue test with three-point bending of annealed specimens at 700 °C ± 5 °C/72 h. The results of fatigue tests applied in practice have a significant impact on the determination of service life, maintenance planning, and overhaul of components made of the IN718 alloy as fatigue damage of parts and components accounts for up to 90% of all fractures and accidents. Thus, knowledge of fatigue life optimizes financial flows in production and maintenance planning, which enables more efficient operation of companies.

## 2. Materials and Methods

### 2.1. Experimental Materials

For the experimental procedures, three Ni-based superalloys were used. Two of them were in the as-cast condition, namely, the former USSR superalloy ZhS6K and Inconel alloy 738C. The third is the well–known Inconel alloy 718. The *SPECTROMAXx* chemical elemental analysis of alloys is shown in Table 1.

**Figure 1:**
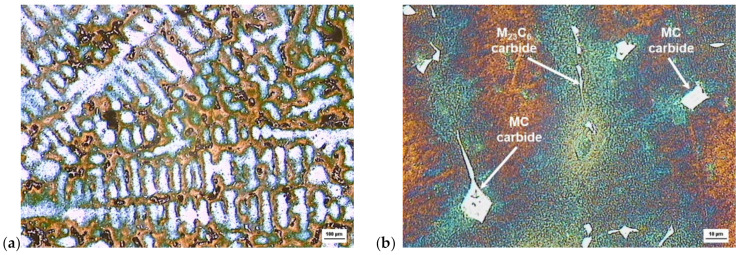
Light microscopy microstructures of the as-cast ZhS6K alloy: (**a**) dendritic segregation; (**b**) carbides at interdendritic areas, etches. Beraha III.

A typical microstructure of the as-cast ZhS6K alloy with significant dendritic segregation and carbides situated in inter-dendritic areas is shown in Figure 1.

Ni-based superalloy ZhS6K was developed in the late 1980s in the former USSR. In the cast state, it is used for turbine blades in the DV-2S aircraft engine. It is a precipitation-hardened alloy, the main precipitation phase being γ′ (Ni_3_Al gamma prime), which is precipitated in the form of fine particles in a supersaturated γ solid solution. The operating temperature range for this alloy is 800–1050 °C. The alloy is produced by the VIM process and is cast from a temperature of 1500–1600 °C, depending on the volume of the casting. It is characterized by good castability with a low shrinkage coefficient of 2–2.5% [38].

**Figure 2:**
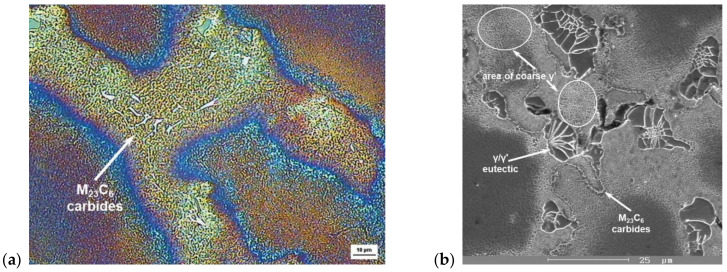
(**a**) Light microscopy microstructure of as-cast IN738C alloy dendritic segregation, carbides in interdendritic areas, etches. Beraha III; (**b**) SEM micrograph showing γ/γ′ eutectic cells, secondary M_23_C_6_ carbides, and areas with coarse γ′ phase situated in interdendritic areas, etches. Marble.

The IN738 alloy is also produced by the VIM process. It is a Ni-based superalloy, precipitation-hardened with high creep resistance at high temperatures and good heat resistance. It is used for the production of turbine components with an operating temperature of up to 980 °C. The mechanical properties of IN738 (tensile strength at elevated temperatures) and the resistance to the negative effects of sulfur at high temperatures were better than those of the more commercially widespread IN713C alloy. Two basic modifications of this alloy are used commercially: a high-carbon alloy designated as IN738C and a reduced-carbon modification designated as IN738LC [39]. Our experimental material is a high-carbon version, IN738C, with the microstructure represented in Figure 2. The microstructure after casting consisted of solid solution elements (Cr, Co, and Mo) in a base nickel matrix–γ phase, eutectic cells of γ/γ′, and after hardening strengthened with the γ′ phase (Ni_3_(Al, Ti)) and M_23_C_6_ carbides at the grain boundaries.

The Inconel 718 alloy was developed from the original Fe-based superalloy by increasing the Ni content and adding the elements Al, Ti, and Nb. Due to the addition of Al and Ti, the intermetallic precipitate γ′-Ni_3_Al (Ll_2_) is precipitated in this superalloy, which, similarly to Ni-based superalloys, is characterized by a high degree of coherence with the basic solid solution γ. However, the main reinforcing phase in this superalloy is the γ″-Ni_3_Nb (BCT DO_22_) phase, which is formed at an average Nb content of about 4–6 wt %. It is a metastable phase that tends to transform to a more stable δ-Ni_3_Nb (orthorhombic lattice) at higher temperatures (above 700 °C) [40,41,42,43,44,45,46]. 

**Figure 3:**
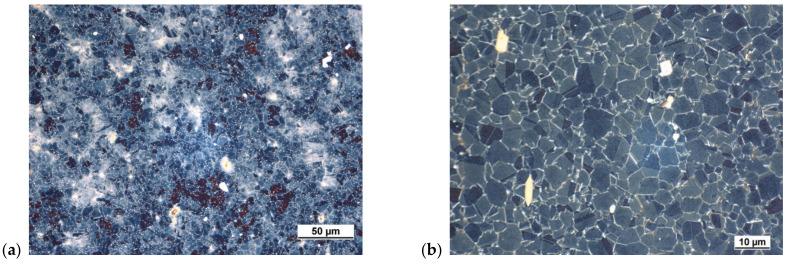
Light microscopy microstructures of wrought IN718 alloy: (**a**) grains in microstructure, according to ASTM E 112/2010 are ASTM12 (5.6 μm); (**b**) crystallization and deformation twins; etches. Beraha III.

It is a weldable superalloy with excellent corrosion resistance at higher temperatures. The field of application of this alloy is up to about 700 °C and is most often used in the form of wrought semifinished products in the aerospace industry. Examples of the microstructure of a wrought IN718 alloy are shown in Figure 3.

### 2.2. Experimental Methods

Specimens from cast Ni-base superalloys ZhS6K and IN738 were additionally heat-treated in an air atmosphere by annealing at 800 °C with different holding times, 10 and 15 h, followed by cooling the samples in air. The wrought Ni-base superalloy IN718 was also treated by additional annealing at 800 °C for 72 h. Annealing, according to available T–T–T diagrams, was chosen to induce significant structural changes that could affect the mechanical properties.

The procedure for the preparation of metallographic specimens consisted of the following steps: cutting with a MTH Micron 3000 metallographic saw (MTH Hrazdil, s.r.o., Brno, Czech Republic); pressing samples in a Struers Cito-Press 1; and grinding and polishing with the Struers Tegra-System (TegraPol-15 and TegraForce-1, STRUERS GmbH, Roztoky, Czech Republic). The final surface treatment of the samples consisted of several steps of sanding with SiC sandpaper no. 320 followed by two-stage polishing of MD-Allegro and OP-S. This procedure was also applied to specimens for light microscopy (LM) and scanning electron microscopy (SEM) and EDS analysis. Transmission electron microscopy (TEM) analysis was carried out in cooperation with the Institute of Materials and Quality Engineering, FMMR, TUKE Košice. Samples for the substructure analysis were processed by standard metallographic methods: sectioned into thin plates and subsequently thinned by mechanical grinding and polishing. The substructure of superalloys was observed by the TEM Jeol JEM-2000FX on the finally thinned foils in a solution of 10% HClO_4_ and 90% CH_3_OH at room temperature and a voltage of 18 V. The identification of the particles of the phases present in the solid solution of superalloys was realized by the selected area electron diffraction (SAED) with the support of CrysTBox software [47]. Light microscopy was used for color etched specimen evaluation (dendrite segregation, grain size, and carbide particles). Light microscopy color etching is useful for the quick identification of dendritic segregation and carbide particles become more pronounced. Additionally, grain boundaries are more visible, which helps in the grain size evaluation.

**Figure 4:**
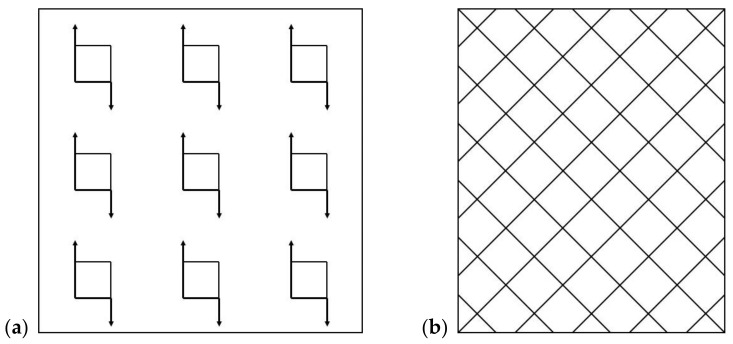
Coherent testing grid for γ′ evaluation. (**a**) Number of γ′ particles; (**b**) volume of γ′ particles.

For phase identification and evaluation, the following methods were used:

- Phase identification was conducted by EDS line analysis and EDS chemical elemental mapping. In some specific situations, EDX analysis was employed.
- The number “N” of γ′-phase and δ-phase particles, a coherent testing grid with nine square shape area probes on SEM micrographs were used (Figure 4a).
- The volume “V” of γ′-phase and δ-phase particles, a coherent testing grid with 50 dot probes made from backslash crossing on SEM micrographs, was used (Figure 4b).

Afterward, the measurement of the values was calculated with Equations (1) and (2). For a detailed description of the methods used, see [48,49,50,51]. Due to long-term heat exposure, the γ′-Ni_3_Al precipitate tends to be coarse. For this reason, the size of the precipitate must be evaluated because a precipitate larger than 0.8 μm decreases the creep resistance of the superalloy and the UTS is significantly reduced.

(1) $$N=1.11\times z^{2}\times x_{\mathrm{mid}}10^{-9}\;\;\;\;\left[{\mu m}^{-2}\right]$$

where N is the number of γ′ particles; z is the magnification used; and x_mid_ is the medium value of γ′-phase measurements.

(2) $$V=\frac{n_{s}}{n}\times 100\;\;\;\;\left[\%\right]$$

where V is the volume of γ′ particles; n_s_ is the medium value of γ′-phase measurement; and n is the number of dot probes (when using a testing grid with 50 dot probes, the equation becomes simpler: V = 2n_s_).

Note that the γ′ phase can be evaluated only in ZhS6K or IN738 cast alloys due to both alloys being strengthened by this precipitating phase. Wrought alloy IN718 is principally strengthened by the γ″ phase and the γ′ phase becomes more visible and coarser after sufficient heat treatment or higher-temperature loading [52]. For the IN718 alloy, we chose the δ-phase evaluation with techniques above-mentioned as the higher content of δ-phase influence consideration on fatigue life change at ambient and high temperatures.

For γ′ phase evaluation, we used five specimens from each alloy ZhS6K and IN738C as well as in three different conditions, starting stage, and two different holding times at 800 °C—first for 10 h and then for 15 h, with cooling on air. The aim was to observe how this heat treatment affects the γ′ volume fraction, size, and distribution. Alloy 718 was not evaluated due to the lack of γ′–phase and hard-to-observe SEM techniques.

Vickers hardness testing was conducted on ZhS6K and IN738 alloys according to the STN EN ISO 6507-1:2006-06 (42 0374) standard. The resulting hardness was established from 10 measurements on each alloy and calculated as a medium value.

**Figure 5:**
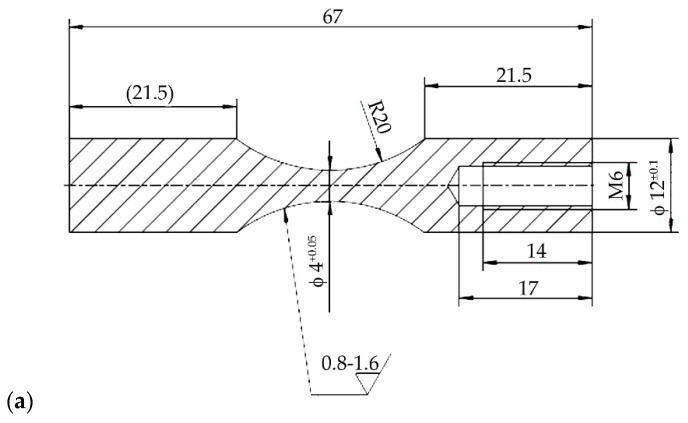

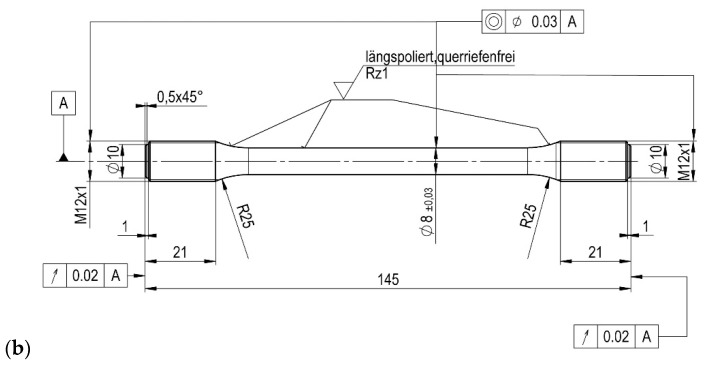
The fatigue test specimens used at room temperature HFFL (**a**) and for LFFL at 700 °C ± 5 °C (**b**).

Due to the expected changes in the microstructure of the wrought Ni-base superalloy IN718 caused by the applied additional annealing and their influence on the mechanical properties, a fatigue test was also performed for this purpose. Samples for fatigue tests at ambient temperature and a temperature of 700 °C ± 5 °C were made by chip machining, turning, from a semi-finished bar. The shape and dimensions of the samples are documented in Figure 5.

High-frequency fatigue tests (HFFL) at ambient temperature were carried out at the KAUP-ŽU experimental facility developed at the Department of Materials Engineering, Faculty of Mechanical Engineering, University of Žilina. The test parameters were as follows: load frequency f ≈ 20,000 kHz, mean load σ_m_ = 0 MPa (symmetrical dynamic load), cycle asymmetry parameter R = −1, and load amplitude varied in the range σ_a_ = 330 ÷ 551 MPa. The value of cycles to fracture N_f_ = 10^8^ and more was determined as run-out.

Low-frequency fatigue tests (LFFL) at 700 °C ± 5 °C were performed on a ZWICK/ROELL Amsler 150HPF 5100 resonant pulsator with a connected furnace with an operating temperature up to 1200 °C. The test parameters were as follows: the test temperature was controlled by ZWICK/ROELL software with a temperature stabilization time of 5 min, load frequency f ≈ 64 Hz, mean load σ_m_ = 0 MPa (symmetrical dynamic load), cycle asymmetry parameter R = −1, and amplitude load varied in the range σ_a_ = 275 ÷ 452 MPa. The value of cycles to fracture N_f_ = 2 × 10^7^ and more was determined as a run-out.

Three-point bending fatigue tests were performed on samples (a simple blocky shape with dimensions 11 × 10 × 55 mm) after annealing at 700 °C/72 h on a ZWICK/ROELL Amsler 150HPF 5100. The test parameters were set as follows: test temperature t = 22 °C, load frequency f ≈ 150 Hz, medium load F_medium_ = −15 kN, the amplitude varied F_a_ = 5.47–10.47 kN. The number of cycles to fracture N_f_ = 2 × 10^7^ was set as run-out. The fatigue life was evaluated by the SN curves.

## 3. Results and Discussion

**Figure 6:**
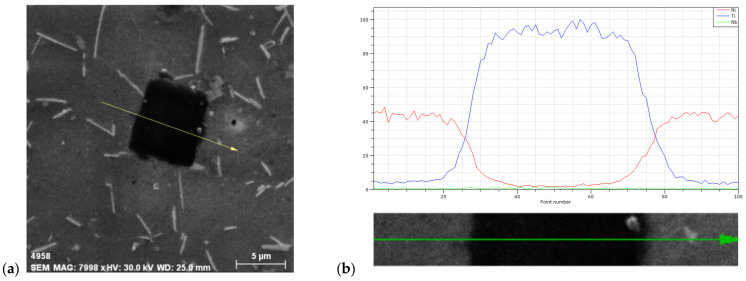
EDS line analysis of carbide phase in alloy IN718: (**a**) overall view; (**b**) line analysis, electrolytic etch. 10 g CrO_3_ + 10 0mL H_2_O.

Light microscopy, as above–mentioned does not always provide satisfactory phase determination in superalloys. This is why we used SEM techniques to obtain a more precise phase identification. There are two methods to determine phases: line analysis and chemical elements mapping. To confirm the presence of carbide particles in the IN718 superalloy, line analysis was used and the results are presented in Figure 6. From this analysis, it is clear to see that the particle is a primary carbide MC type, namely NbC or TiC; in this particular case, it was a TiC primary carbide type.

**Figure 7:**
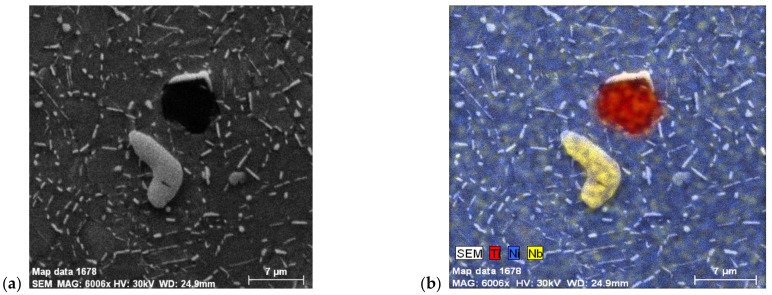
EDS chemical elements mapping, alloy IN718: (**a**) overall view; (**b**) carbide particle identification, etch. Marble.

To confirm this, chemical mapping was used. In Figure 7a,b, it can be seen that even carbides rich in Nb content are light gray (in EDS mapping they are yellow), rounded particles, and carbides rich in Ti content are sharp-edged, dark gray particles (in EDS mapping they are red.

**Figure 8:**
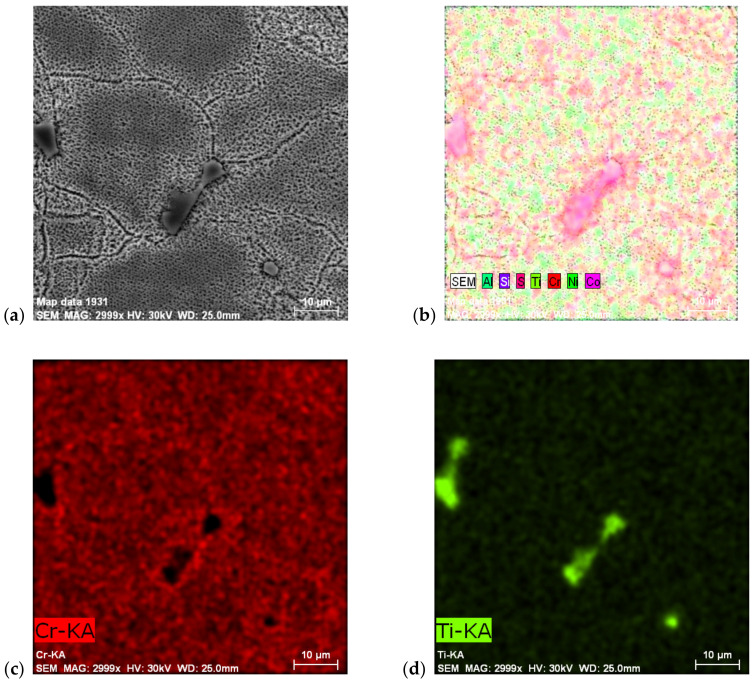
Ni-based superalloy ZhS6K: (**a**) SEM microstructure of grain boundaries and evaluated carbide particle; (**b**–**d**) EDS chemical elements mapping, etches. Marble.

The same procedures were used for the cast Ni-based ZhS6K superalloy used (see Figure 8), with even chemical element distribution in cross-section. These cast superalloys are typical for their chemical heterogeneity, represented by significant dendritic crystallization. Figure 8a shows an overall view of the microstructure, where dark areas are paths of primary dendritic areas, which crystallized as first during solidification. This area is characterized by a very fine γ′-Ni_3_Al phase (the Ti content in this strengthening phase is questionable due to the high correlation of Ti to carbon; therefore, Ti mainly goes to primary carbides, TiC) interestingly, no carbide was present in this area. All carbides, regardless of whether they were primary MC or secondary M_23_C_6_, were situated in interdendritic areas when coarse γ′-Ni_3_Al was also present. Figure 8b,c shows the distribution of the elements, with Figure 8d showing the primary MC type carbide, TiC.

Superalloys are strengthened through three principal mechanisms: (a) solid-solution hardening (SSH); (b) precipitation hardening (PH); and (c) oxide-dispersion strengthening (ODS). For the superalloys discussed in this article, the first two strengthening mechanisms are the most important. For that reason, only they will be described further.

Precipitation hardening of the solid solution is one way of increasing the strength of the matrix by adding alloying elements that dissolve in the matrix in various ways. Because these additional alloying elements have a different atomic radius than the basic element (Ni), they cause deformation of the crystallographic lattice and thus make the dislocation movement more difficult. The greater the difference in atomic radius between the alloying element and the basic element forming the solid solution, the higher the effect of precipitation hardening, but not more than the difference in the radii of atoms of about 10% [11]. The use of high-melting-point alloying elements stabilizes the lattice of the base solid solution and retards diffusion, especially at higher temperatures. This principle is used in precipitation hardening using the γ′-Ni_3_Al phase. By excluding the γ′-Ni_3_Al precipitate from the supersaturated solid solution γ, the stacking fault energy (SFE) is significantly reduced and thus the dislocation slip in the alloy is prevented, which is essentially the main deformation mechanism of superalloys at high temperatures [53]. Thus, the reduction in the stacking fault energy (SFE) complicates the movement of dislocations, especially their change in propagation direction and its jump into another slip system [2]. In materials that crystallize in face-centered cubic lattices (FCC), as is the case with Ni-based superalloys, a decrease in the stacking fault energy results in the disintegration of the dislocation structure (long dislocations are divided into shorter ones), the formation of new hexagonal-close packed (HCP) phases with intrinsic SFE, and thus an increase in the energy of the dislocation transition from the phases crystallized in the FCC lattice to the phases crystallized in the HCP lattices.

Another mechanism for increasing the strength of solid solutions is consolidation through a cluster of atoms, or by arranging the atoms over a short-range. This strengthening mechanism is related to the suitable arrangement of atoms in the electron orbitals of specific elements (e.g., Mo, W, Cr, Al, and Re). These elements, dissolved in the basic Ni matrix, achieve a higher strengthening effect than if they were dissolved in an Fe, Ti, Co, or V matrix [11]. This mechanism of strengthening (i.e., using a cluster of atoms) was observed in a monocrystalline superalloy CMSX-2 alloyed with Re, where clusters of Re atoms of about 1 nm were observed, which significantly limited the movement of dislocations and thus increased the strength of the alloy. The strengthening of the basic solid solution by arranging the atoms over a short-range and its effect decreases at temperatures around 60% of the melting point (0.6T_M_) as a consequence of the improving diffusion conditions. The diffusion and improved conditions at temperatures around 0.6T_M_ are responsible for the significant deformation and reduction in creep strength in alloys that are strengthened by the hardening of the basic solid solution (SSH) [53,54]. As reported by Sims et. al. [14], increasing the strength by strengthening the basic solid solution is achieved up to about 815 °C. The elements that provide SSH are Al, Fe, Ti, Cr, W, and Mo. Alloying with higher density elements is used to reduce the diffusion rate. However, the disadvantage is the increase in the density of the superalloy and the tendency to form topologically close phases (TCP).

Increased creep resistance at high temperatures is achieved in Ni-based superalloys by precipitation hardening (PH). Precipitation hardening of Ni-based superalloys is provided by elements such as Al, Ti, and Nb. These elements are characterized by having limited solubility in the Ni matrix, which decreases with decreasing temperature. Subsequently, by employing heat treatment and precipitation, fine, dispersed precipitates are precipitated in the supersaturated solid solution γ.

These intermetallic precipitates are coherent phases of γ′-Ni_3_Al, Ti, or γ″-Ni_3_Nb and prevent dislocation motion. Dislocations in such heat-treated structures can move in only two ways: crossing or passing precipitates. Donachie et al. and Sims et al. [2,14] suggest that the effectiveness of PH can be assessed by three basic qualities:

- The coherence stress between the matrix γ and the precipitates γ′/γ″, which arises from the difference in the size of the lattice parameters;
- The energy of the antiphase boundary (APB) in the vicinity of the excluded ordered phases γ′ and γ″. The energy of the antiphase boundary, APB, characterizes the dislocation energy required for the dislocation to cross the precipitate. Such overcutting of the precipitate very often leads to a loss of coherence between the matrix and the precipitate; and
- The volume fraction of precipitate γ′, γ″, and the particle size of these precipitates.

The latter factor, the volume fraction of γ′-Ni_3_Al precipitates and the particle size, were evaluated using coherent testing grid methods. In this way, only cast superalloys ZhS6K and IN738C in which the γ′-phase was visible when observed by SEM were evaluated; the IN718 alloy in the initial state contains a very difficult to observe γ′-phase, which becomes more visible only after additional heat treatment at temperatures above 700 °C (see Figure 10). As already mentioned, the volume fraction of the γ′-phase significantly affects the mechanical and high-temperature properties of superalloys. Donachie et. al. [2] stated that the average size of the precipitate γ′ should be in the range of 0.35–0.45 μm, which will ensure satisfactory creep resistance. They also limited the size of carbide particles in their work, which should not exceed approximately 5 μm due to their negative effect as initiators of fatigue failure. Another negative effect of high-temperature exposure and annealing at high temperatures is the formation of TCP phases such as σ-phase or Laves phases, which occur in superalloys with a high content of Cr and Fe in the temperature range 750–800 °C. The results of the γ′-phase evaluation (volume fraction, particle size, and the number of particles) are shown in Table 2. 

**Figure 9:**
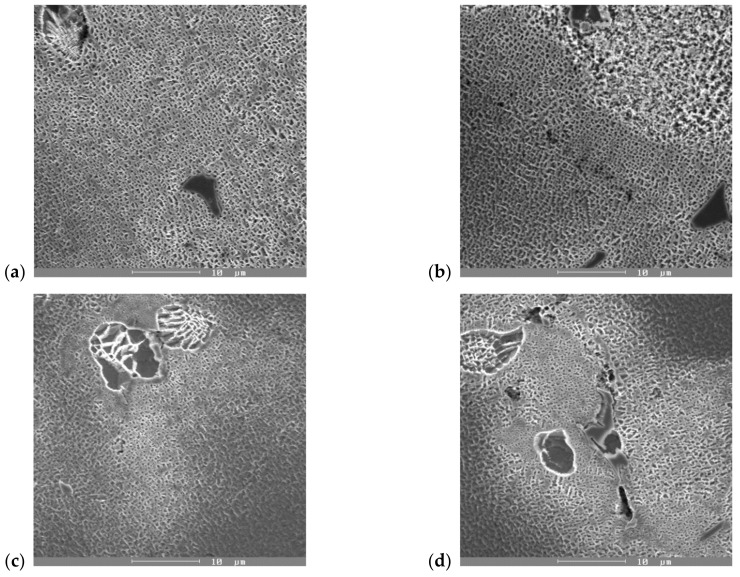
Micrographs of γ′-phase: (**a**) starting stage; (**b**) after 800 °C/10 h AC of ZhS6K alloy; (**c**) starting stage; and (**d**) after 800 °C/10 h AC of IN738C alloy, SEM, etches. Marble.

Analyzing the results presented in Table 2, it follows that annealing the alloys at 800 °C for 10 or 15 h caused an increase in the volume fraction of the γ′-phase. The most significant increase in the volume fraction of the γ′-phase was seen in the ZhS6K alloy. From the original value of V = 39.4%, it increased to 72.4% (staying at an annealing temperature for 10 h) and 76.6% (staying at an annealing temperature for 15 h), which represents an overall increase in the volume fraction of the γ′-phase by 83.7% or 94.4%, respectively (see Figure 9a,b) compared to the as-cast state. The increase in the volume fraction of the γ′-phase was also associated with an increase in the linear dimension u of this phase from the original value of 0.61 μm to 0.69 μm (or 0.72 μm with a dwell time of 15 h). The alloy IN738 did not show such a significant increase in volume V of the γ′-phase; it changed from 60.4% in the initial state to 66.6% or 71.2%, respectively, which represents an increase in the volume of 10.26% (for 10 h) or 17.88% (for 15 h) (see Figure 9c,d). The change in the linear dimension of the γ′-phase in the IN738 alloy was negative (i.e., the dimensions decreased), which was due to the precipitation of new volumes of the fine γ′-phase and not the coarsening of the original phase.

Vickers hardness tests were performed to determine how the mechanical properties of cast Ni superalloys change due to applied annealing. An increase in hardness is also likely to increase the strength characteristics such as ultimate tensile strength (UTS) and yield stress of said superalloys. Based on the obtained hardness measurement results, which are shown in Table 3, it can be stated that the applied annealing increased the hardness of the investigated superalloys. In the case of the IN738 superalloy, the highest increase in hardness was recorded with a holding time of 15 h at the annealing temperature. The hardness increased from 393 HV10/10 to 413 HV10/10 compared to the starting stage, which represents an increase of approximately 5% in hardness. The increase in hardness is related to the increased volume fraction V of the γ′-phase (see Table 3) and the decrease in the size of the precipitate. The most significant increase in hardness was again found in the case of the ZhS6K superalloy after a holding time of 15 h at the annealing temperature specifically, from 403 HV10/10 at the starting stage to 493 HV10/10, which represents an increase in hardness of approximately 23%. Such a significant increase in hardness is a consequence of the re-precipitation of new volumes of the hardening γ′-phase, the volume fraction V of which increased by almost 95% compared to the starting state. The increased volume fractions of the γ′-phase in the case of both superalloys were caused by suitable diffusion conditions at a given annealing temperature, and the chosen annealing can be considered as the “next step” in precipitation hardening, which corresponds to the measurement results in Table 2.

**Figure 10:**
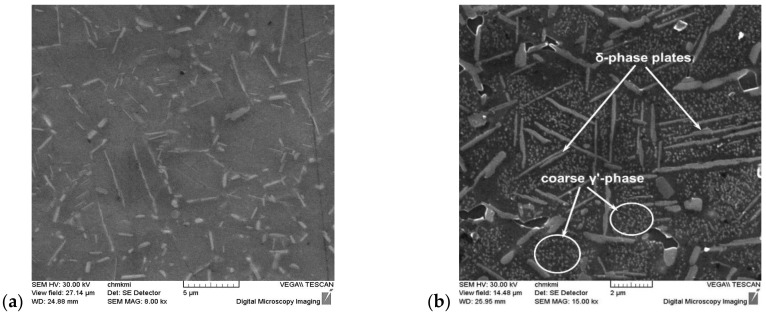
SEM micrographs of IN 718 alloy compared the starting stage (**a**) to post heat-treatment at 700 °C/72 h (**b**). Mind the coarse γ′-phase and higher volume of δ-phase plates at grain boundaries and inside grains. Etches. 10 g CrO_3_ + 100 mL H_2_O (2 V, 1 s, 90 mA/cm^2^).

**Figure 11:**
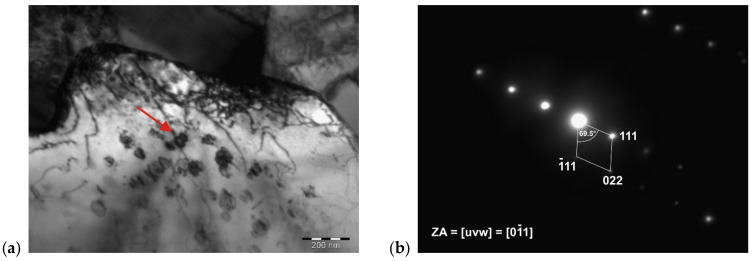
Detail of γ′-Ni_3_(Ti, Al) phase particles (**a**) and diffraction patterns of one of the particles (**b**).

**Figure 12:**
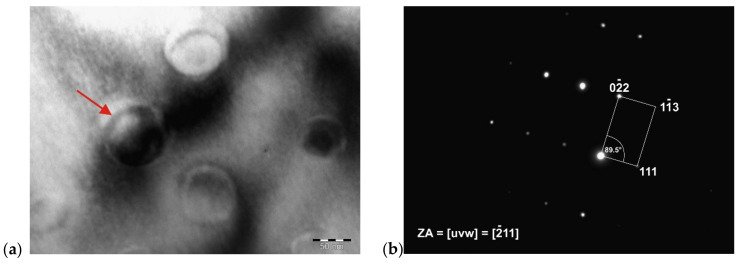
Detail of γ′-Ni_3_(Ti, Al) phase particles (**a**) and diffraction pattern of one of the particles (**b**).

**Figure 13:**
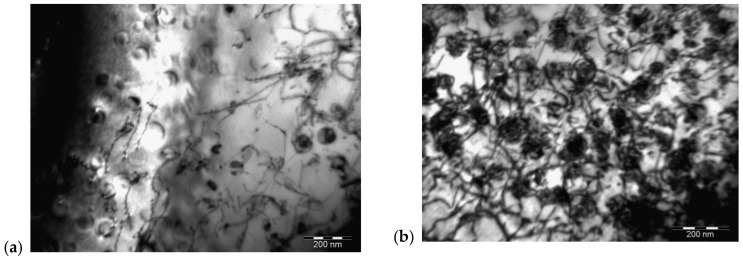
(**a**) Particles present in the solid solution of the IN718 alloy in an annealed state at various magnifications and (**b**) interaction of these particles with the dislocation net.

SEM analysis of IN718 alloy after annealing at 700 °C/72 h (see Figure 10) confirmed an increased occurrence of the precipitating γ′-phase. To identify this phase, a TEM analysis was also performed, which confirmed that it is a γ′-phase with a different morphology of exclusion. The results of the TEM analysis are documented in Figure 11, Figure 12 and Figure 13.

Figure 11 illustrates the γ′-Ni_3_(Ti, Al) phase particles present in a solid solution of the IN718 alloy, which has been identified based on a spot diffraction pattern. As can be seen, the γ′-Ni_3_(Ti, Al) phase particles have a semicoherent interface with the matrix and their average size was 48.5 nm. Evaluation of diffraction patterns obtained by SAED in TEM with the support of CrysTBox software confirmed the presence of γ′-Ni_3_(Ti, Al) phase particles (FCC crystallographic system, Pm3m-221 space group, parameter a = 3.561 Å). The zone axis, the indexed reflections of crystallographic planes, and the angle between the indexed reflections (φ = 69.5°) are clear in Figure 11b.

Figure 12 shows the globular particles of the γ′-Ni_3_(Ti, Al) phase in a solid solution of IN718 alloy, which has been identified based on the spot diffraction pattern. The average size of these particles was 65.4 nm. The FCC crystal lattice, Pm3m space group, and the parameter a = 3.561 Å were confirmed. Figure 12b illustrates the zone axis, the indexed reflections of the crystallographic planes, and the angle between the indexed reflections (φ = 89.5°).

Based on previous analyses, it can be argued with high probability that semi-coherent γ′-Ni_3_(Ti, Al) phase particles were present in the solid solution of the IN718 alloy in the annealed state, which is visible in Figure 13 and also shows the interaction of these particles with dislocations.

The increase in strength by age-hardening is achieved by the arrangement and coherent stresses between the γ′-phase and the matrix γ, which is related to the size of the precipitate γ′. The larger the size of the precipitate and the higher the volume fraction, the greater the age-hardening effect, as dislocations are forced to cross these precipitates (Figure 13). However, this effect is limited by the so-called Orowan′s dislocation propagation mechanism [55], where a significant coarsening of the precipitate γ′ forms wider channels between them and thus dislocations can be propagated by bypassing the coarse precipitates and reducing the effect of precipitation hardening [52]. The resulting shape of the coherent precipitate is influenced, on one hand, by the elastic stress, which is related to the value of the parameter of the matrix (FCC)-precipitate (L1_2_) lattice mismatch and also to the interfacial energy of the matrix–precipitate [56]. The value of the mismatch parameter between γ/γ′ is closely related to the chemical composition, especially the content of elements such as Ni, Al, Cr, Co, Ti, or Mo, which are dissolved in the aforementioned phases with different at % concentrations. Dodaran et al. [57] noted changes in the at % fraction of elements: Al from 2.56 at % and 5.6 at %, where the Al content in the γ′-phase changes from 8.58 at % to 12.25 at %; Ti of 2.0 at % and 5.5 at %, where the Ti content in the γ′-phase changes from 8.5 at % to 12.7 at %; Cr from 9.0 at % and 26.5 at %, where the Cr content in the γ′-phase changes from 1.06 at % to 1.67 at %; and Co from 14.2 at % and 36.0 at %, where the Co content in the γ′-phase changes from 5.1 at % to 16.25 at %. They further stated that the elements Ti, Cr, and Mo in the ternary system Ni–Al–X dissolve in substitution and tend to replace Al atoms in the Ni_3_Al γ′-phase. Co also dissolves in the Ni–Al–Co ternary system by substitution, but tends to replace the Ni atoms in the Ni_3_Al γ′-phase. Thus, changes in the chemical composition also affect the lattice parameters of the matrix γ and γ′-phase. This also changes the value of the lattice mismatch, which is calculated according to Equation (3) [58,59]:

(3) $$\delta =\frac{2\left(a_{\gamma \prime }-\;a_{\gamma }\right)}{\left(a_{\gamma \prime }+\;a_{\gamma }\right)}$$

where δ is the value of the lattice mismatch; a_γ_ is the lattice parameter of the matrix γ; and a_γ′_ is the lattice parameter of the γ′-phase. As stated in Goodfellow et al. [59] where the influence of the Mo content on the value of the lattice mismatch is discussed, with rising at % Mo, the lattice parameters of the γ and γ′-phase matrix increased, but the value in the lattice mismatch parameter decreased to about 4 at % Mo. At higher Mo contents, the value of the lattice mismatch can even be negative. An example of the change in the lattice parameters of the γ, γ′-phase, γ″-phase, and the resulting value of the lattice mismatch δ (%), depending on the temperature and the heat treatment method, is given in Table 3 [60].

Of course, these changes in the chemical composition of the γ′-phase are also related to the stacking fault energy value (SFE). Yang et al. [61] stated that the elements Cr, Co, Ti, and Mo, with increasing concentration, decreased the matrix γ SFE. In the case of the γ′-phase, the SFE, Co, and Cr with increasing concentration decreased its value and Ti and Mo with increasing concentration increased the SFE value. Dou et al. [62] found the same effect of alloying elements on SFE and stated that, in terms of the creep rupture strength, the Co content, which reduces SFE in both the γ and γ′-phase, had the most significant effect. Tian et al. [63] dealt with the influence of the Co content on the change in SFE and the related change in the creep rupture strength. They stated that when the Co content rose from 5 wt % to almost 23 wt %, the SFE value dropped from 40.1 mJ·m^−2^ to 24.9 mJ·m^−2^. The decrease in the SFE value of the alloy thus simplified the dissociation dislocation in the γ matrix, supported the γ′-phase micro-twinning process, increased the creep resistance, and improved the creep properties. In the case of a low value of the parameter of the mismatch between the matrix and precipitate gratings, a precipitate of a predominantly globular shape is formed (Figure 12a). With the increasing value of the lattice mismatch parameter, cell precipitates of more complex geometric shapes are formed. The globular form of the precipitate is based on the general theory of nucleation and crystal growth of a new phase (precipitate) in the solid phase. In equilibrium nucleation, the newly formed phase tends to reduce the value of free enthalpy ΔG according to Equation (4):

(4) $$\Delta G=\;-V\Delta G_{V}+{A\gamma }_{I}+V\Delta G_{S}$$

where ΔG is the value of free enthalpy associated with the phase transformation; V is the volume of the nucleus; ΔG_V_ is the volume free enthalpy; A is the area of the nucleus γ_I_ is the interfacial energy; and ΔG_S_ is the change in strain energy. Therefore, to start the nucleation of the precipitate, it is necessary to overcome the so-called activation barrier ΔG_act._, which is associated with fluctuations in temperature and chemical composition. The nuclei of the new precipitate are characterized by homogeneous nucleation, where for homogeneous nucleation to take place, the condition of significant subcooling of the system and a small critical radius of the nucleus must be met. Based on this condition, it is clear that the morphology of the resulting γ′-phase precipitate in the matrix γ is globular to reduce the total ΔG value (Equation (4)). These precipitates have the highest degree of coherence in the initial stage of formation and the lowest value of mismatch (see Table 3 for direct aging δ_γ/__γ′_). During the growth of the precipitate (coarsening), there is a gradual loss of coherence.

The mechanical properties of the precipitation-hardened superalloys changed significantly at temperatures that are just below the dissolution temperatures of the precipitates. This fact is in good agreement with [54] where the mechanical properties of the alloy IN 718 decreased significantly at a temperature of about 700 °C, which represents the dissolution temperature and transformation of the metastable γ″-Ni_3_Nb phase to a stable δ-Ni_3_Nb phase [2]. In the same way, the high-temperature characteristics of this alloy change as a result of the said transformation and the dissolution of the precipitate. 

To fulfil the secondary goal, how the increased δ-phase formation affects the fatigue properties, the δ-phase volume evaluation at the starting stage and after annealing at 700 °C ± 5 °C was undertaken (Table 4).

**Figure 14:**
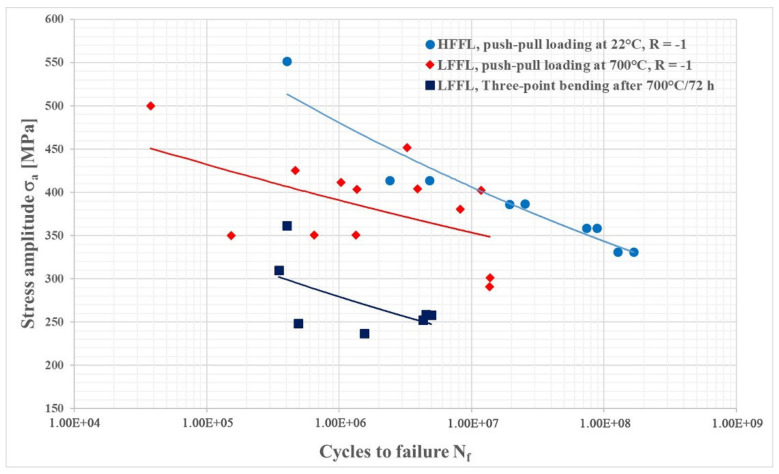
The S-N curves of fatigue life for IN718.

The fatigue properties of the IN718 superalloy in the temperature range of about 700 °C showed a similar character to the mechanical properties in terms of tensile strength (UTS) and yield strength. The results of the fatigue test at 700 °C ± 5 °C, and after three-point bending of annealed samples at room temperature interpreted using the S–N curves are shown in Figure 14. 

The obtained fatigue life values were compared with the results of the fatigue test at an ambient temperature of about 22 °C ± 5°C, but the loading frequency, in this case, was f = 20,000 kHz. Fatigue failure and fatigue life are affected by several factors such as the formation of oxides, which form at high temperatures at the surface and affect the mechanism of fatigue crack closure by excluding phases such as BCT DO_22_ Ni_3_Nb γ″-phase and orthorhombic D0_a_ Ni_3_Nb δ-phase, as demonstrated in Ling et al. [64]; the frequency of loading and the number of cycles to fracture, as reported by Song et al. [32]; or the heat treatment method, as reported by Zhao et al. [65].

**Figure 15:**
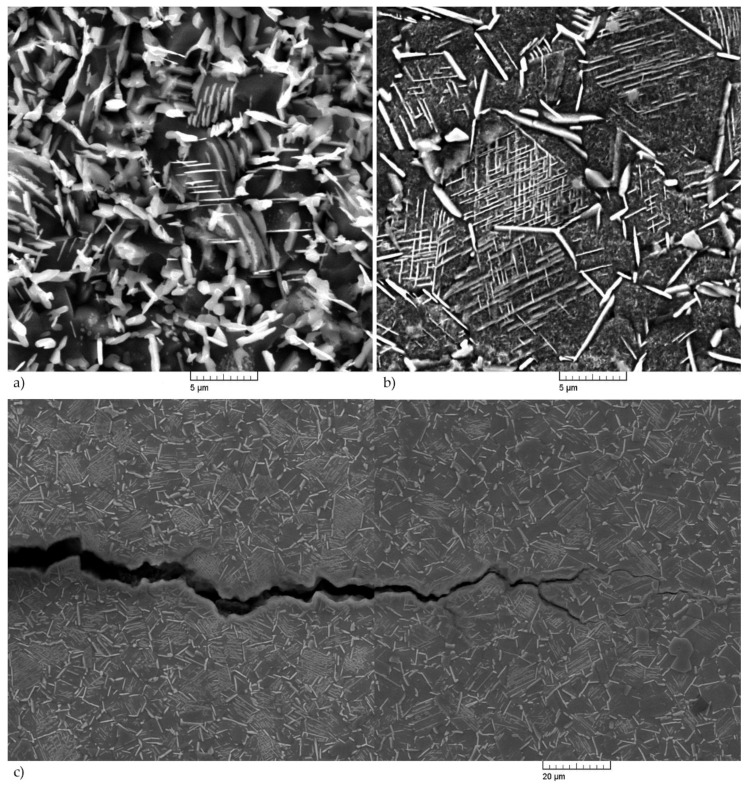
The δ-phase volume fraction after annealing at 700 °C ± 5 °C/72 h (**a**) and fatigue test at 700 °C ± 5 °C (**b**); fatigue crack propagation with the Widmanstätten δ-phase close to the surface and different secondary fatigue crack propagation mechanism (**c**).

The results of HFFL fatigue tests performed on samples at ambient temperature showed that the load amplitude when reaching the number of cycles to fracture N_f_ = 1.68 × 10^8^ and with the proportion of excluded δ-phase, V = 39.5%, σ_a_ = 330 MPa. The results of LFFL tests on samples at 700 °C ± 5 °C with a fraction of δ-phase, V = 65%, showed a load amplitude σ_a_ = 275 MPa at a run-out of N_f_ = 2 × 10^7^, which represents an approximately 20% decrease in fatigue compared to the results obtained at ambient temperature. However, the difference in fatigue life when comparing approximately the same values of the number of cycles to fracture N_f_ ≈ 2 × 10^7^ was even greater and represents a difference of up to 40% (σ_a_ = 386 MPa at N_f_ = 1.9 × 10^7^, comparison with the result at ambient temperature). These results show that increasing the proportion of δ-phase in the alloy from the original 39.5% to 65% results in a significant reduction in fatigue life. Results of the fatigue test at three-point bending were even lower. Of course, the reason is also the different loading mode at three-point loading, but results support the fact that increasing δ-phase volume fraction decreases the fatigue life (the difference was almost 95%, σ_a_ = 386 MPa at 1.9 × 10^7^ for push–pull at room temperature to σ_a_ = 197 MPa at 2.0 × 10^7^ for three-point bending of annealed specimens). Fractographic analysis of the samples after the fatigue test showed a different nature of fatigue crack propagation. This crack propagated in a mixed-mode by either a transcrystalline or intercrystalline mechanism. The mode of propagation is influenced by the orientation of the original grains concerning the applied load and, of course, by the presence of the precipitated δ-phase, which predominantly had the morphology of the Widmanstätten needles located inside the grains (Figure 15). The decrease in fatigue life is due to the increased proportion of the δ-phase and the increased dislocation density (see Figure 10b and Figure 13b) in the sample tested at 700 °C.

In SSH alloys in which elements such as Mo and W have limited solubility, the importance of carbides lies in the high-temperature strengthening of grain boundaries [52]. This consolidation mechanism increases the creep strength when, at higher temperatures, the creep processes take place mainly at grain boundaries.

**Figure 16:**
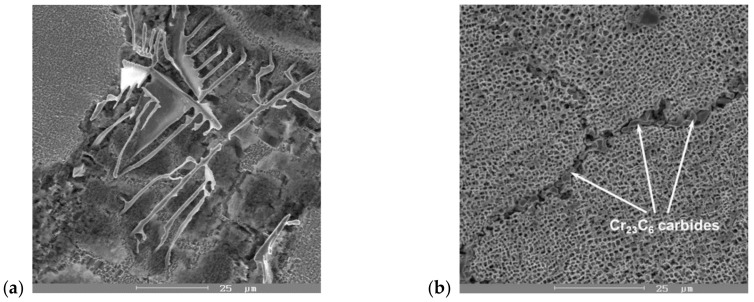
Types of carbides presented in superalloy microstructure with higher carbon content: (**a**) primary TiC carbide “Chinese” script like in ZhS6K alloy; (**b**) secondary Cr_23_C_6_-type carbide at the grain boundary of ZhS6K alloy, SEM, etches. Marble.

Carbides precipitated at grain boundaries slow down grain boundary slippage and redirect creep into the grains where diffusion is slower. The highest effect of grain boundary strengthening is achieved by discontinuous, chain-separated carbides. Primary carbides are described by the relationship MC or M_6_C, where “M” represents Mo, W, Ti, and Nb. These carbides of MC (Figure 16a) or M_6_C type, when exposed to long-term heat exposure, tend to change to secondary carbides of the M_23_C_6_ type, as do Cr carbides. Since heat-resistant alloys must contain a high Cr content to ensure their heat resistance at high temperatures, it is almost impossible to avoid the formation of complex Cr_23_C_6_ secondary carbides (Figure 16b).

The formation of large amounts of Cr carbides is an undesirable phenomenon. Their formation significantly depletes the basic solid solution of Cr and thus reduces the corrosion stability of the grain boundaries as these carbides are mainly situated at the grain boundaries.

As these carbides, precipitated in the form of a carbide network at the grain boundaries, are brittle, they represent a site of the frequent initiation and growth of cracks, thereby reducing the mechanical properties (UTS, YS) and ductility of the alloy. Ideally, they are in a discontinuous, chain-like arrangement, where these carbides are excluded as fine particles with a small size and not a single large particle [52]. Due to exposure to high temperatures, carbides containing Mo and W can also form at grain boundaries. Their formation results in a reduction in the content of these elements in the matrix and thus creates a depleted area around the grain boundaries [66]. Paradoxically, the depleted area may increase the creep resistance of the material. The creep process depends on the temperature and diffusion rate at the grain boundaries. The higher the temperature, the higher the diffusion rate, the more active the slip systems are at the grain boundaries. The carbides Mo and W situated at the boundaries block the slip at the grain boundaries and transfer the creep processes from the grain boundary to the grain, where the diffusion is slower. Thus, by slowing down the diffusion and transferring the creep process to the inside of the grains, the creep resistance increases. In the case of the exclusion of carbides as dispersed particles inside the grain, these carbides have the same effect as intermetallic precipitates. However, their main task remains to strengthen the grain boundaries, thus preventing slippage at the grain boundary and increasing creep resistance [1].

Materials such as Ni-base superalloys, methods, and technologies were recognized several years ago as new ones, today, they often seem to not be efficient enough when compared to the market and industrial requirements, especially in the area of the additive manufacturing of stationary or jet turbine components. To meet the challenges of the Industry 4.0 philosophical knowledge of a fundamental relation between the microstructure and mechanical properties of Ni-base superalloys are needed. In this context, new practical and scientific results are of great interest and importance to mechanical engineering. Special attention is continuously given to the problems of new manufacturing technologies and modern conceptions of manufacturing systems [67,68] that allow us to make high-quality products with a high level of effectiveness.

Based on the experiments and their results, we can highlight several facts. First, comprehensive microstructure analysis of cast ZhS6K and IN738C superalloys and wrought IN718 superalloy was performed using available techniques such as color etching and DIC to increase the contrast of dendritic segregation of cast superalloys. This is a fundamental method for the rapid identification of dendritic segregation, applicable not only in the study of Ni-base superalloys, but also to the whole spectrum of cast materials such as Ti, Al, and Mg alloy. When evaluating γ′-phase precipitates, we used the SEM fundamental method of quantitative evaluation of volume fraction (V in %) and the number of phase particles per area (N in µm^−2^), which can be successfully used in the evaluation of structural components if the software of the optical microscope does not allow for the correct thresholding of particles (i.e., the phases are only in grayscale). For more advanced analysis of particles than carbides, the EDS line and EDS chemical elements mapping method were used to distribute the chemical elements in the matrix or particles, which also involves the basic equipment of a SEM microscope. Of course, the most accurate analysis of parts including diffractograms is provided by TEM. The possibilities of TEM analysis are limited only by the number of modules purchased in the microscope evaluation software.

A separate step consists of fatigue tests, which are demanding not only in terms of the instrumentation, but also concerning the complexity of the whole process of fatigue Ni superalloys. In our case, we used two different approaches to fatigue testing. Both tests were performed only on the IN718 superalloy due to time constraints and access to experimental material. Fatigue tests on cast superalloys could not be performed since the real turbine blades of aircraft engines were microstructurally analyzed and not the model alloy. Fatigue tests of the alloy IN718 were carried our at ambient temperature, 22 ± 5 °C at a load frequency of about 20,000 kHz and a temperature of 700 ± 5 °C and a load frequency of about 65 Hz, in both cases with a parameter of cycle asymmetry R = −1 (symmetrical push–pull). Another fatigue test, three-point bending, was performed on samples after annealing 700 °C/72 h at an ambient temperature of 22 ± 5 °C with a load frequency f ≈ 150 Hz and a cycle asymmetry parameter R > 1. This load method is more complex and combines bending and tensile load, which corresponds to significantly lower values of fatigue life compared to the classic push–pull load. The obtained results are unique in that most authors [32,64,65] have performed fatigue tests either only at ambient temperature and with a low load frequency or with a different parameter of the cycle asymmetry R. The data on increased δ-phase volume fraction and its influence on fatigue life have not been studied up until now. For this reason, we considered the results of high-temperature fatigue and its comparison with the fatigue life obtained at ambient temperature to be unique, especially if they were made on materials with the same grain size and the same processing method (i.e., the effect of changing the grain size), so a different γ′/γ″ phase ratio was eliminated.

## 4. Conclusions

The subject of the study of changes in microstructural components γ′/δ after the application of heat treatment were two cast superalloys ZhS6K and IN738C, in which the change in γ′-phase morphology and the effect of decreasing chemical heterogeneity, represented by SDAS factor, on Vickers hardness change were evaluated. The effect of increased δ-phase volume fraction on the change in dynamic fatigue characteristics with symmetrical cyclic loading with cycle asymmetry parameter R = −1 and with load frequency ≈ 20,000 kHz was evaluated on the wrought IN718 superalloy, which was additionally heat-treated as well as cast superalloys, at ambient temperature and with a load frequency ≈ 64 Hz at a temperature of 700 °C ± 5 °C. The conclusions from the performed experiments are:

- The basic solid solution of the elements in the Ni, γ-matrix crystallizes in the FCC lattice and the elements Co, Fe, Cr, Mo, and W are predominantly substitutionally dissolved in it. All the mentioned elements decreased the SFE of the γ matrix and increased the creep properties of the alloys.
- The intermetallic precipitate γ′-Ni_3_(Al, Ti), crystallizes in the L1_2_ lattice and is formed mainly by the elements Al and Ti. It is a coherent precipitate with the matrix γ, and the elements Al and Ti can be partially replaced by the elements Nb, Ta, and Cr. It is the main hardening phase in cast IN738C and ZhS6K alloys precipitated in the form of globular or cubic particles.
- The Vickers hardness increased in both cast Ni-based superalloys (IN738C and ZhS6K) after the applied annealing at 800 °C/for 10–15 h, respectively. The increase in expression was 5% for IN738C and 23% for ZhS6K superalloys, which is closely related to the increasing volume of the fine γ′-phase fraction as a result by diffusion re-distribution of alloying elements from previously heterogeneous dendritic segregation.
- The microstructure of IN718 alloy is formed by austenitic γ matrix, MC carbides, and strengthening phases γ″, γ′, with 39.5% of δ-phase in the starting stage.
- The δ-phase was identified with SEM and TEM diffraction and is situated on grain boundaries or twin boundaries in the plate (needle) like shape and blocky shape presented intergranularly, respectively.
- Annealing at 700 °C/72 h resulted in an increase in the volume fraction of the δ-phase to 52.5%. Said increase in volume is due to the transformation of the metastable γ″-phase when this phase releases Nb, which subsequently forms new δ-phase volumes. Furthermore, γ′-phase coarsening was observed, which has the effect of reducing the coherence of the precipitate with the γ matrix.
- During the LFFL fatigue tests at 700 °C ± 5 °C, additional volumes of δ-phase are formed due to the mechanical load and an increase in volume fraction of up to 65% was observed; δ-phase transforms into the Widmanstätten pattern, whish influences negatively on fatigue crack propagation, causing intercrystalline cleavage.
- It is clear from the S–N fatigue life curves that increasing the volume fraction of the δ-phase from 39.5% to 65% will reduce the fatigue life value by 40% at push–pull loading (from σ_a_ = 386 MPa at room temperature to σ_a_ = 275 MPa at 700 °C) and even more, up to 95% at three-point bending (from σ_a_ = 386 MPa at room temperature σ_a_ = 197 MPa for three-point bending load of annealed specimens at 700 °C/72 h).
- Based on the fatigue tests, it can be stated that the exclusion of thee δ-phase across grain boundaries in the blocky or plate-like forms up to 39.5% generally had a positive effect on the mechanical properties. It stabilizes grain boundaries and reduces creep rate and slippage at grain boundaries and does not significantly affect the dynamic fatigue properties of IN718. However, increasing the δ-phase volume to 65% due to a combination of high temperature and dynamic loading caused its transformation into Widmanstatten needles, which significantly reduced the fatigue service life of the IN718 alloy at high temperatures.
- In terms of the application of fatigue life results in practice, usable, especially in the field of maintenance and overhaul planning of components made of IN718 alloy, operating in different load conditions and temperatures (up to 700 °C), experiments have shown that approximated and recalculated component life at the symmetrical load with the cycle asymmetry parameter R = 1 is at an approximate load frequency of 64 Hz and comparable σ_a_ = 275 MPa, with a δ-phase content of 39.5% and an ambient temperature of approx. 8463 h and at a test temperature of 700 ± 5 °C, with a δ-phase content of 65% at approx. 87 h. In the case of annealed specimens and three-point bending loads with the cycle asymmetry parameter R > 1 at ambient temperature with σ_a_ = 197 MPa, frequency 150 Hz, with a δ-phase content of 52.5%, the fatigue life was approx. 38.82 h. From the above interpretation also concerning the fatigue loading mode used, the negative influence of the excluded δ-phase on the dynamic properties of the IN718 alloy was obvious.
- Various forms of carbides, which are formed at a carbon content of 0.02–0.2% and contain elements such as Ti, Ta, Hf, Nb, Cr, Mo, and W. Primary carbides MC decompose due to heat treatment and service and form additional forms of carbides such as M_23_C_6_ and M_6_C that occur at grain boundaries. The precipitated carbide phases have a predominantly positive effect on the mechanical properties of superalloys.

## 5. Possible Future Research in the Field

In subsequent research, we would like to focus on fatigue tests of wrought Ni superalloys IN718, IN625, and Nimonic 80A and steels of the AISI group and Ti alloys with a focus on the most commercially successful Ti64 alloy. Our workplace, the Department of Materials Engineering, University of Žilina, has a ROTOFLEX device for performing fatigue tests at rotation bending load, with a cycle asymmetry parameter of R = −1 and a load frequency of ≈60 Hz; another available test machine is the ZWICK/ROELL AMSLER 150 HFP 5100. This equipment uses the resonance principle with a constant or variable amplitude and mean load. The device allows us to perform fatigue tests with the parameter of cycle asymmetry R = −1 (push-pull) at ambient temperature, or in the temperature range −70 °C ÷ 1200 °C, or with R < 1 (three-point or four-point bending) with variable frequency loads up to 150 Hz. In the case of fatigue tests with three-point bending, it is possible to perform tests on samples after different methods of controlled heat treatment. Again, a comprehensive structural analysis will be performed on the samples using all available techniques (color etching, deep etching, DIC, SEM, EDS, and mapping) and a thorough TEM analysis will be performed in cooperation with the Institute of Materials and Quality Engineering, FMMR, TUKE Košice before and after fatigue tests including a fractographic analysis of the fracture surfaces.

## Figures and Tables

**Table 1:** The SPECTROMAXx chemical analysis (in wt. %) of alloys used in the experimental work.

Alloy	Elements, wt. %											
	C	Co	Cr	Al	Ti	Nb	W	Mo	Fe	Mn	Ta	Ni
Cast superalloys *												
ZhS6K	0.202	4.76	12.44	5.22	3.05	0.012	5.28	3.48	0.192	0.002	-	Bal.
IN738C	0.17	8.5	16.0	3.4	3.4	0.9	2.6	1.7	0.05	0.02	1.75	Bal.
Wrought superalloy **												
IN718	0.026	0.14	19.3	0.57	0.96	5.3	-	2.99	Bal.	0.07	0.01	53.32

Notes: * For cast superalloys, the Ni content is given for balance; ** For wrought superalloy, the Fe content is given for the balance.

**Table 2:** Results for γ′-Ni_3_Al phase evaluation in various conditions.

Alloy (Condition)	Number of γ′-Phase N (μm^−2^)	Volume Fraction of γ′-Phase V (%)	The Size of γ′-Phase u (μm)	Hardness (HV 10)
Alloy 738C				
Start stage	1.68	60.4	0.76	393
10 h/air	1.63	66.6	0.64	410
15 h/air	1.58	71.2	0.67	413
**Alloy ZhS6K**				
Start stage	2.47	39.4	0.61	403
10 h/air	1.50	72.4	0.69	490
15 h/air	1.49	76.6	0.72	494

**Table 3:** Lattice parameters (nm) of phases γ, γ′, and γ″ and mismatch value δ (%) for alloy IN718.

Heat Treatment	Lattice Parameters “a” and Mismatch “δ”				
	a_γ_	a_γ′_	a_γ__″_	δ_γ__/__γ′_	δ_γ__/__γ__″_
Direct aging	0.35907	0.35904	0.35761	0.08412	0.32121
950 °C AC + aging	0.36019	0.35899	0.35874	0.33384	0.34511
1000 °C AC + aging	0.36163	0.36097	0.35877	0.18254	0.63827
1080 °C AC + aging	0.36166	0.36044	0.35882	0.33868	0.75209

**Table 4:** Results of δ-phase volume measurement by the quantitative metallography method.

Alloy IN718	The Volume of δ-Phase Fraction V [%]
Starting stage	39.5
Annealing at 700 °C ± 5 °C/72 h	52.5
Fatigue test at 700 °C ± 5 °C	65

## Data Availability

Data sharing not applicable.
